# Genetic diversity of *Helicobacter pylori* type IV secretion system *cagI* and *cagN* genes and their association with clinical diseases

**DOI:** 10.1038/s41598-023-37392-7

**Published:** 2023-06-24

**Authors:** Yasaman Azizimoghaddam, Sadaf Kermanpour, Nasrin Mirzaei, Hamidreza Houri, Ali Nabavi-Rad, Hamid Asadzadeh Aghdaei, Abbas Yadegar, Mohammad Reza Zali

**Affiliations:** 1 Foodborne and Waterborne Diseases Research Center, Research Institute for Gastroenterology and Liver Diseases, Shahid Beheshti University of Medical Sciences, Tehran, Iran; 2 Basic and Molecular Epidemiology of Gastrointestinal Disorders Research Center, Research Institute for Gastroenterology and Liver Diseases, Shahid Beheshti University of Medical Sciences, Tehran, Iran; 3 Gastroenterology and Liver Diseases Research Center, Research Institute for Gastroenterology and Liver Diseases, Shahid Beheshti University of Medical Sciences, Tehran, Iran

**Keywords:** Bacteria, Bacteriology, Clinical microbiology, Microbial genetics

## Abstract

A number of *cag*PAI genes in the *Helicobacter pylori* genome are considered the most evolved genes under a diversifying selection and evolutionary pressure. Among them, *cagI* and *cagN* are described as a part of the two different-operon of *cag*PAI that are involved in the T4SS machinery, but the definite association of these factors with clinical manifestations is still unclear. A total of 70 *H. pylori* isolates were obtained from different gastroduodenal patients. All isolates were examined for the presence of primary *H. pylori* virulence genes by PCR analysis. Direct DNA sequence analysis was performed for the *cagI* and *cagN* genes. The results were compared with the reference strain. The *cagI*, *cagN*, *cagA*, *cagL*, *vacA* s1m1, *vacA* s1m2, *vacA* s2m2, *babA2*, *sabA*, and *dupA* genotypes were detected in 80, 91.4, 84, 91.4, 32.8, 42.8, 24.4, 97.1, 84.3, and 84.3% of the total isolates, respectively. The most variable codon usage in *cagI* was observed at residues 20–25, 55–60, 94, 181–199, 213–221, 241–268, and 319–320, while the most variable codon usage in CagN hypervariable motif (CagNHM) was observed at residues 53 to 63. Sequencing data analysis of *cagN* revealed a hypothetical hexapeptide motif (EAKDEN/K) in residues of 278–283 among six *H. pylori* isolates, which needs further studies to evaluate its putative function. The present study demonstrated a high prevalence of *cagI* and *cagN* genes among Iranian *H. pylori* isolates with gastroduodenal diseases. Furthermore, no significant correlation between *cagI* and *cagN* variants and clinical diseases was observed in the present study. However, all patients had a high prevalence of *cag*PAI genes including *cagI*, *cagN*, *cagA*, and *cagL*, which indicates more potential role of these genes in disease outcome.

*Helicobacter pylori* (*H. pylori*) is a Gram-negative, microaerophilic bacterium that can chronically colonize the human stomach. This recalcitrant pathogen infects more than 50% of the world’s population and is considered the primary cause of chronic active gastritis, gastric and duodenal ulcers, mucosa-associated lymphoid tissue (MALT) lymphoma, and gastric adenocarcinoma^1,2^. *H. pylori* infection is recognized as the main risk factor for the development of gastric cancer, which is the fifth most common malignancy and the third leading cause of cancer-associated morbidity worldwide^3^. The severity of *H. pylori*-induced gastric disorders seems to be associated with several parameters, including host genetic polymorphism, host inflammatory responses, environmental factors, and bacterial virulence genotype^4,5^.

*H. pylori* is associated with high genetic variability including virulence genes due to genetic plasticity, rearrangement of DNA, and high transformation and recombination frequency. Thus, *H. pylori-*infected patients exhibit different patterns of disease progression and clinical outcomes geographically. To date, several virulence factors coding genes have been identified in the genome of *H. pylori* such as *cagA*, *vacA*, *babA*, *sabA*, and *dupA*^4,6^. CagA oncoprotein is the best-studied virulence-associated factor of *H. pylori* that is translocated into the host gastric epithelial cells via the type 4 secretion system (T4SS). The *H. pylori* T4SS machinery is encoded by a gene cluster that comprises an approximately 40 kb chromosomal region named *cag* Pathogenicity Island (*cag*PAI)^7,8^. *cag*PAI encodes about 27–31 genes, by which a subset of these genes encodes the main components of the T4SS apparatus spanning bacterial membranes. Moreover, about 15 to 16 different proteins of the T4SS are required for the translocation of CagA and peptidoglycan fragments into the host cell^9^. Upon translocation, CagA modulates the host cell signaling pathways which ultimately results in the loss of membrane polarity, cell elongation, secretion of inflammatory cytokines, and development of gastric adenocarcinoma^10^. *cag*PAI encodes several unique Cag components that have no sequence similarities to any other bacterial proteins involved in T4SS. However, a number of *cag*PAI genes such as *cagI* and *cagN* were proposed to be the most probably evolved genes under a diversifying selection and evolutionary pressure^11^. CagI, a 41.5 kDa protein encoded by the *cagI* (*cag19*/*hp0540*) gene, does not share any sequence and topological homology with any other known proteins^12,13^. On the other hand, CagN (Cag17/HP0538), a 32–35 kDa protein encoded by the *cagN* gene (*hp0538*), is a poorly characterized component of the T4SS that appears to be localized to the bacterial inner membrane rather than the periplasm^9,12,14,15^.

There are conflicting reports regarding the precise role of CagI and CagN in CagA translocation, IL-8 secretion from gastric epithelial cells, and *H. pylori* T4SS machinery^14,16–20^. Recent studies have revealed that CagI is involved in the pilus biogenesis of T4SS and is essential for CagA translocation by binding to β1 integrins of the host cell^21,22^. On the other hand, the deletion of *cagN* can reduce the phosphorylation degree of CagA in the host cell and it is not considered a substrate for the T4SS^14^. However, the putative role of CagI and CagN in CagA translocation and *H. pylori* pathogenesis is yet to be fully elucidated. The oncogenic potential of *H. pylori* strains is associated with their virulence capacity, genetic diversity, and specific sequence polymorphisms within the key genes involved in the translocation and phosphorylation of T4SS effectors^23–26^. Therefore, we aimed to determine the prevalence of *cagI* and *cagN* genes and their amino acid sequence polymorphisms in Iranian *H. pylori*-infected patients with various gastroduodenal diseases. We further investigated the probable association between the genetic variants of *cagI* and *cagN* and other virulence genotypes of *H. pylori* with clinical consequences.

## Materials and methods

### *H. pylori* clinical isolates and biopsy specimens

Gastric biopsy specimens were obtained from 70 patients who underwent upper gastroduodenal endoscopy at the Research Institute for Gastroenterology and Liver Diseases in Tehran between January 2017 and May 2019. Three antral biopsies were taken from each patient and immediately placed in transport media containing Thioglycolate supplemented with 3% yeast extract (Oxoid Ltd., Basingstoke, UK) and 1.3 g/L agar (Merck, Germany). All patients provided written informed consent. The study was approved by the Institutional Ethical Review Committee of the Research Institute for Gastroenterology and Liver Diseases at Shahid Beheshti University of Medical Sciences (Project No. IR.SBMU.RIGLD.REC.1398.023). All methods were performed in accordance with the relevant guidelines and regulations.

### *H. pylori* culture and identification

Biopsy specimens were carefully homogenized and inoculated onto Brucella agar plates (Merck, Germany) supplemented with 7% (v/v) horse blood, 10% fetal calf serum (FCS), Campylobacter-selective supplement (vancomycin 2.0 mg, polymyxin 0.05 mg, trimethoprim 1.0 mg), and amphotericin B (2.5 mg/l). The incubation was performed at 37 °C for 3–7 days under a microaerophilic atmosphere (5% O_2_, 10% CO_2_, and 85% N_2_) in a CO_2_ incubator (Innova® CO-170; New Brunswick Scientific, USA). The suspected colonies were identified as *H. pylori* based on colony morphology, Gram staining, positive reaction for oxidase, catalase, as well as urease tests, and also by *H. pylori* gene-specific PCR following the previously described protocols^27,28^. Pure cultures from confirmed isolates were kept in 0.5 ml of brain heart infusion (BHI) medium (Merck, Germany) containing 15% glycerol plus 20% FCS, and stored at − 80 °C until further analysis.

### Genomic DNA extraction

Genomic DNA was extracted from freshly harvested colonies on agar plates, using the QIAamp DNA Mini Kit (QIAGEN, Hilden, Germany) according to the manufacturer’s instructions. The quality of DNA was checked by using a NanoDrop® ND-1000 spectrophotometer (Thermo Fisher Scientific, USA). The extracted DNA samples were stored at − 20 °C until PCR assay.

### Genotyping of *H. pylori* virulence-associated genes

PCR analysis was performed to detect virulence target genes including *cagL*, *cagA*, *vacA* alleles (s1/s2 and m1/m2), *babA2*, *sabA*, and *dupA* genes using specific primers (Table S1). Briefly, PCR mixtures in a volume of 25 µl consisted of 2 µl of template DNA (approximately 200 ng), 0.1 mM of each primer, 2.5 µl of a tenfold concentrate PCR buffer, 100 mM of deoxynucleotide triphosphates, 1 mM MgCl_2_, and 1.5 U of Super-Taq™ DNA polymerase (HT Biotechnology Ltd., Cambridge, UK). PCR amplifications were performed in a thermocycler (Eppendorf, Hamburg, Germany) under the following conditions: initial denaturation at 94 °C for 4 min, followed by 30 cycles of denaturation at 94 °C for 1 min, annealing at the indicated temperature for each reaction in Table S1 for 45 s, extension at 72 °C for 1 min. A final extension step was performed at 72 °C for 10 min to ensure the full extension of the PCR products. PCR amplicons were electrophoresed on a 1.2% TBE agarose gel, stained with ethidium bromide, and examined under a UV transilluminator. *H. pylori* J99 (CCUG 47,164) and a no-template mixture served as positive and negative controls in each PCR experiment, respectively.

### Primer designation for *cagI* and *cagN* genotyping

The NCBI GenBank database (http://www.ncbi.nlm.nih.gov/genbank/) and the DNA Data Bank of Japan (http://www.ddbj.nig.ac.jp/) were searched for all available complete and partial *cagI* and *cagN* sequences of *H. pylori* strains. Based on pairwise and multiple nucleotide sequence alignments of *cagI* and *cagN* genes from different *H. pylori* strains and using the complete relevant sequence of *H. pylori* P12 (CP001217.1) as the reference strain, two pairs of specific primers were designed from the conserved regions for detection of complete related sequences using CLC Sequence Viewer 8 software (https://www.qiagenbioinformatics.com/). The selected primer target sites were compared to all available complete and partial *cagI* and *cagN* sequences of *H. pylori* strains with the Basic Local Alignment Search Tool (http://blast.ncbi.nlm.nih.gov/Blast.cgi).

### Analysis of *cagI* and *cagN* diversity by PCR sequencing

For DNA sequencing of *cagI* and *cagN*, PCR amplification was carried out in a final volume of 25 µl using designed specific primers including 5′-CATTTGACTTACCTTGATTAC-3′ (*cagI*-F) and 5′-TTTGAGCACTTGTTGGTTGG-3′ (*cagI*-R), 5′-GAGCGACAAAACAACTATGC-3′ (*cagN*-F) and 5′-GATCCCTAGAACAAAGTAAGC-3′ (*cagN*-R) yielding DNA fragments of about 1377 and 1192 bp in length, respectively. The PCR products were purified using the Silica Bead DNA Gel Extraction Kit (Thermo Scientific, Fermentas, USA) followed by sequencing on both strands using an automated sequencer (Macrogen, Seoul, Korea). DNA sequences were edited by Chromas Lite version 2.5.1 (Technelysium Pty Ltd, Australia) and BioEdit version 7.2.5^29^. The *cagI* and *cagN* nucleotide and amino acid sequences were aligned to *H. pylori* strain P12 as a reference strain (GenBank: CP001217.1). The single nucleotide variations and codon usage of the sequences were examined using BioEdit version 7.2.5.

### Phylogenetic analysis

Phylogenetic trees were generated for CagI and CagN nucleotide and amino acid sequences using Molecular Evolutionary Genetics Analysis version 7.0 (MEGA7)^30^. The evolutionary history was inferred by the Maximum Likelihood trees using the Tamura 3-parameter model and Poisson correction method for nucleotide and amino acid sequences, respectively.

### Nucleotide sequence accession numbers

The complete and partial nucleotide sequences of *cagI* and *cagN* genes from *H. pylori* strains determined in this study were deposited in the NCBI GenBank database under the accession numbers MG573078-MG573107 (*cagI*) and MG559675-MG559720 (*cagN*).

### Statistical analysis

The statistical associations between *H. pylori* virulence genotypes and different clinical statuses were determined by the Chi-square and Fisher’s exact tests. A two-sided *P* value of less than 0.05 was regarded as statistically significant. The IBM SPSS Statistics for Windows version 21.0 (Armonk, NY: IBM Corp.) was used for all statistical analyses.

### Ethics approval and consent to participate

This work deals with clinical bacterial strains isolated from human gastric biopsies. No tissue material or other biological material was stored from the patients, only subcultured bacterial isolates. Informed consent was obtained from all individual participants included in the study. All procedures performed were following the ethical standards retrieved from the Institutional Ethical Review Committee of the Research Institute for Gastroenterology and Liver Diseases (RIGLD) at Shahid Beheshti University of Medical Sciences (Project No. IR.SBMU.RIGLD.REC.1398.023).

## Results

### Demographic and clinical characteristics of patients

The median age of the patients was 45.6 years (ranging from 14 to 75 years). Of the study cohort, 32.8% (*n* = 23) were male and 67.2% (*n* = 47) were female. According to the endoscopic and histopathology findings, 39 patients (55.7%) were diagnosed with non-ulcer dyspepsia (NUD), 23 patients (32.9%) had peptic ulcer disease (PUD), 7 patients (10%) had intestinal metaplasia (IM), and 1 patient (1.4%) had gastric cancer. Three patients (4.3%) suffered from gastritis and duodenitis simultaneously. Table S2 indicates the demographic characteristics and clinical status of the included subjects. From each of the 70 cases, *H. pylori* were isolated by culture, and the isolates were approved by detection of the *glmM* and 16S rRNA genes.

### Virulence genotypes and variants

The molecular analysis revealed that the *cagA*, *cagI*, *cagN*, *cagL*, *vacA* s1m1, *vacA* s1m2, and *vacA* s2m2 positive strains had a prevalence of, respectively, 84% (*n* = 59), 80% (*n* = 56), 91.4% (*n* = 64), 91.4% (*n* = 64), 32.8% (*n* = 23), 42.8% (*n* = 30), and 24.4% (*n* = 17) while *babA2*, *dupA*, and *sabA* were detected in, respectively, 97.1% (*n* = 68), 84.3% (*n* = 59), and 84.3% (*n* = 59) of the isolates included in this investigation (Table 1). There was no statistically significant association between the *H. pylori* virulence genotypes and the clinical status of the patients (*P* > 0.05). In the present study, 100% (23/23) of the PUD and 94.9% (37/39) of the NUD strains were positive for the *babA2* gene by PCR. Furthermore, the prevalence of *cagN* and *cagL* genes for PUD strains is attributed to 95.6% (22/23) and 91.3% (21/23), respectively. In the meantime, patients suffering from NUD showed a frequency of 89.7% (35/39) and 94.9% (37/39) for the same genes as PUD. When it comes to *vacA* allelic combinations, *vacA* s1m2 was found to be the most common allele among the strains recovered from the PUD patients (52.2%), whereas 42.8 and 33.3% of allelic combinations were assigned to *vacA* s1m1 and *vacA* s2m2, within the IM and NUD strains, respectively.

**Table 1:** Distribution of virulence genotypes in relation to clinical status among 70 *H. pylori* strains.

Virulence genotypes	Clinical status				Total (*n* = 70)
	NUD (*n* = 39)	PUD (*n* = 23)	IM (*n* = 7)	GC (*n* = 1)	
*cagI*-positive	32 (82%)	17 (73.9%)	6 (85.7%)	1 (100%)	56 (80%)
*cagN*-positive	35 (89.7%)	22 (95.6%)	6 (85.7%)	1 (100%)	64 (91.4%)
*cagA*-positive	33 (84.6%)	19 (82.6%)	6 (85.7%)	1 (100%)	59 (84.3%)
*cagL*-positive	37 (94.9%)	21 (91.3%)	5 (71.4%)	1 (100%)	64 (91.4%)
*vacA* s1m1	12 (30.8%)	8 (34.8%)	3 (42.8%)	0 (0%)	23 (32.8%)
*vacA* s1m2	14 (35.9%)	12 (52.2%)	3 (42.8%)	1 (100%)	30 (42.8%)
*vacA* s2m2	13 (33.3%)	3 (13%)	1 (14.3%)	0 (0%)	17 (24.3%)
*babA2*-positive	37 (94.9%)	23 (100%)	7 (100%)	1 (100%)	68 (97.1%)
*sabA*-positive	32 (82%)	20 (87%)	6 (85.7%)	1 (100%)	59 (84.3%)
*dupA*-positive	32 (82%)	20 (87%)	6 (85.7%)	1 (100%)	59 (84.3%)

*GC* gastric cancer, *IM* intestinal metaplasia, *NUD* nonulcer dyspepsia, *PUD* peptic ulcer disease.

### *cagI* variants in patients with different clinical status

Out of 56 *cagI*-positive *H. pylori* strains, the *cagI* gene of 30 strains was randomly selected and sequenced. The full-length *cagI* gene was successfully sequenced in 27 *H. pylori* strains. Moreover, the *cagI* gene was partially sequenced in three strains due to poor-quality of sequence data or sequencing errors. According to our sequencing data, there was no insertion or deletion in the full-length *cagI* fragment from 27 *H. pylori* studied, and sequence alignments were therefore straightforward. In addition, we performed in-frame translation for the *cagI* gene into amino acid sequences and investigated rates and locations of CagI variants. The distribution of amino acid polymorphisms in CagI of *H. pylori* strains is represented in Fig. S1 and Table 2. The most variable codon usage was observed at residues G20–I25, Q55–E60, G94, M181–A199, K213–T221, and Q241–A268. As we expected, the SKVIVK hexapeptide motif (376–381) located at the C-terminal of CagI was completely conserved among the *cagI*-sequenced *H. pylori* strains.

**Table 2:** The frequency of amino acid substitutions of CagI among clinical strains of *H. pylori* (*n* = 27) from patients with different clinical status.

Residue^a^	Reference	Variant	NUD (*n* = 19)	PUD (*n* = 7)	IM (*n* = 1)
2	K	N	1	–^b^	–
3	C	S/F	1/1	–	–
6	S	D	1	–	–
9	S	F	1	–	–
12	T	I	1	–	–
22	E	G	1	3	–
23	V	A/I	1/1	3/1	–
25	I	M	2	1	–
34	I	N	1	–	–
40	A	V	1	–	–
44	T	A	1	–	–
51	A	V	2	–	–
57	N	S	9	5	1
94	G	S	10	6	1
116	A	G	1	–	–
162	A	T	1	–	–
166	A	V	–	1	–
182	E	K	1	–	–
187	A	T	1	–	–
190	S	N	–	1	–
192	S	F	1	–	–
195	A	T	2	1	–
199	A	T	1	–	–
204	G	S	1	–	–
213	K	E	3	–	–
221	T	E	4	–	–
243	A	T	4	2	–
246	A	V	–	1	–
254	S	N	2	–	–
257	A	T	1	–	–
262	I	F	1	–	–
263	E	Q	1	–	–
268	A	V/E	5/1	1/–	1/–
305	D	G/N	–/1	1/1	–
319	G	E	1	2	–
320	E	Q	1	2	–
351	L	F	1	–	–
353	K	T	1	–	–
368	T	M/K	1/1	–	–
375	S	G	–	2	1

*NUD* nonulcer dyspepsia, *PUD* peptic ulcer disease, *IM* intestinal metaplasia.

^a^Positions of amino acid residues correspond to the *H. pylori* P12 reference strain.

^b^Positions of amino acid residues similar to the *H. pylori* P12 reference strain.

### *cagN* variants in patients with different clinical status

Regarding *cagN* sequence analysis, 46 strains were randomly sent for direct DNA sequencing from 64 *cagN*-positive *H. pylori* strains. The complete *cagN* gene was successfully sequenced in 43 *H. pylori* strains. Furthermore, the *cagN* gene fragments of three strains were partially sequenced for the same reasons as the *cagI* gene. The *cagN* sequencing findings showed a high level of variability in CagN nucleotide and protein sequences. The most variable codon usage was observed at residues 53 to 63, the so-called CagN hypervariable motif (CagNHM). Moreover, a hypothetical hexapeptide (EAKDEN/K) was inserted in residues 278–283 among six *H. pylori* strains. Interestingly, this motif was observed two times in a row in one of these clinical strains (EAKDENEAKDEN). The other insertion sequences were detected between residues 224–225 and 234–235 for KV and KN amino acids in one of the strains. The sequencing data analysis revealed that these insertion sequences in the *cagN* gene caused no frameshift mutations as compared to the P12 reference strain. Figure S2 and Table 3 showed the distribution of amino acid polymorphisms of CagN among 43 *H. pylori* strains in this study.

**Table 3:** The frequency of amino acid substitutions of CagN among clinical strains of *H. pylori* (*n* = 43) from patients with different clinical status.

Residue^a^	Reference	Variant	NUD (*n* = 24)	PUD (*n* = 14)	IM (*n* = 4)	GC (*n* = 1)
8	L	I	–	1	–^b^	–
15	S	F	2	–	–	–
17	V	A/I	3/1	1/1	–	–
18	I	V	11	7	2	–
32	S	N	–	1	–	–
33	E	K	1	–	–	–
36	E	K	9	1	2	–
38	A	V	24	14	4	1
39	A	V	–	1	–	–
46	K	T	–	1	1	–
48	L	F	8	6	1	1
49	H	Y	7	4	1	1
52	H	R	–	1	–	–
53	G	D	24	14	4	1
54	D	N	1	–	–	–
55	E	K	7	3	–	–
57	I	V	16	10	2	–
59	E	K	17	13	3	–
61	E	K	3	–	–	–
63	K	E	16	12	3	–
80	A	V	–	2	1	–
98	V	I	18	9	1	1
102	A	V	2	–	–	–
103	A	T/S	8/–	5/1	1/–	–
106	K	R	3	1	–	–
114	I	T	3	6	–	–
117	T	N/H	7/14	3/9	3/1	1/–
118	P	S	–	1	–	–
121	D	N	2	–	–	–
125	S	G	3	2	–	–
129	A	T	16	11	4	1
134	N	H	2	–	–	–
137	D	G	1	–	–	–
140	D	N	2	2	–	–
148	E	G	13	6	2	–
149	A	S	7	4	2	–
154	A	T/V	2/1	4/–	1/–	1/–
155	A	T	–	1	–	–
160	N	D	18	11	4	–
161	E	K	–	1	–	–
170	I	V	3	–	–	–
174	C	G	1	–	–	–
182	D	N	–	1	1	–
191	G	D	–	2	1	–
194	D	E	1	–	–	–
199	A	T/V	5/–	5/1	2/–	–
203	E	K	1	–	1	–
208	I	V	1	–	–	–
221	S	N	2	–	–	–
224	K	R	1	–	–	–
225	L	F	1	–	–	–
226	A	V	1	–	–	–
227	L	F	–	2	1	1
228	N	H	–	1	–	–
232	N	S	–	1	–	–
233	R	K	–	1	–	–
241	T	A	22	13	4	1
248	K	R	–	1	–	–
259	T	I	1	–	–	–
262	A	T	2	1	–	–
263	S	G	–	–	1	–
264	K	E	23	14	4	1
267	T	A	15	11	2	1
268	T	A	1	12	–	–
273	N	S	1	–	–	–
279	T	A/V	7/1	5/–	2/–	1/–
280	F	S	1	1	1	–
284	R	H	4	2	–	–
285	S	F/P	2/–	–	–/1	–
287	S	F	1	1	–	–
288	E	D	1	–	–	–
302	A	V	1	–	–	–
304	E	G	24	14	4	1

*NUD* nonulcer dyspepsia, *PUD* peptic ulcer disease, *IM* intestinal metaplasia, *GC* gastric cancer.

^a^Positions of amino acid residues correspond to the *H. pylori* P12 reference strain. The inserted sequences are not indicated in the table.

^b^Positions of amino acid residues similar to the *H. pylori* P12 reference strain.

### Phylogenetic analysis of *H. pylori* CagI and CagN

**Figure 1:**
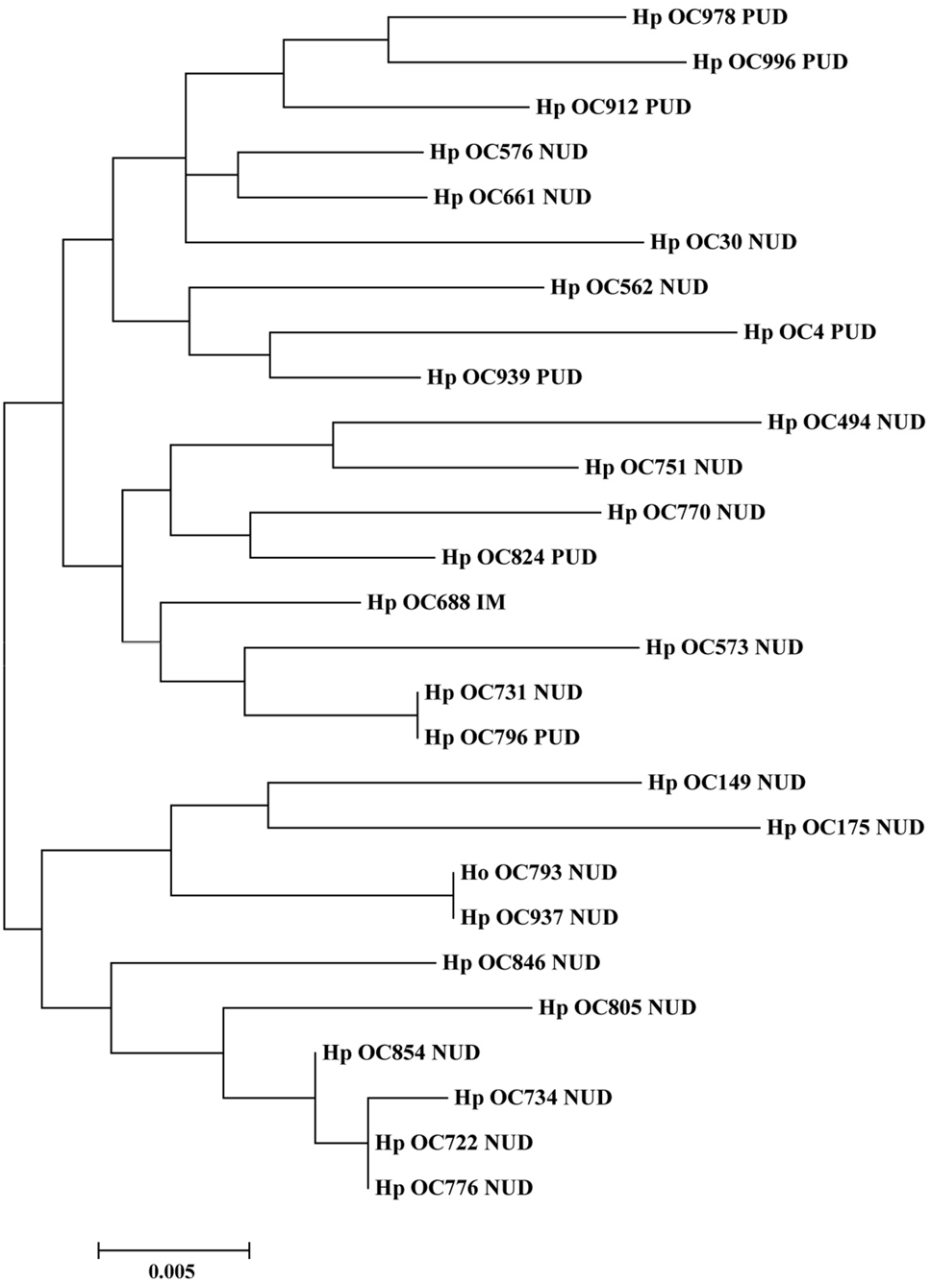
Phylogenetic tree of *H. pylori* clinical strains (*n* = 27) based on *cagI* nucleotide sequences. The maximum likelihood tree of concatenated sequences was constructed using MEGA7 software with the bootstrap method at 1000 replications. The evolutionary distances were computed using the Tamura 3‐parameter model.

**Figure 2:**
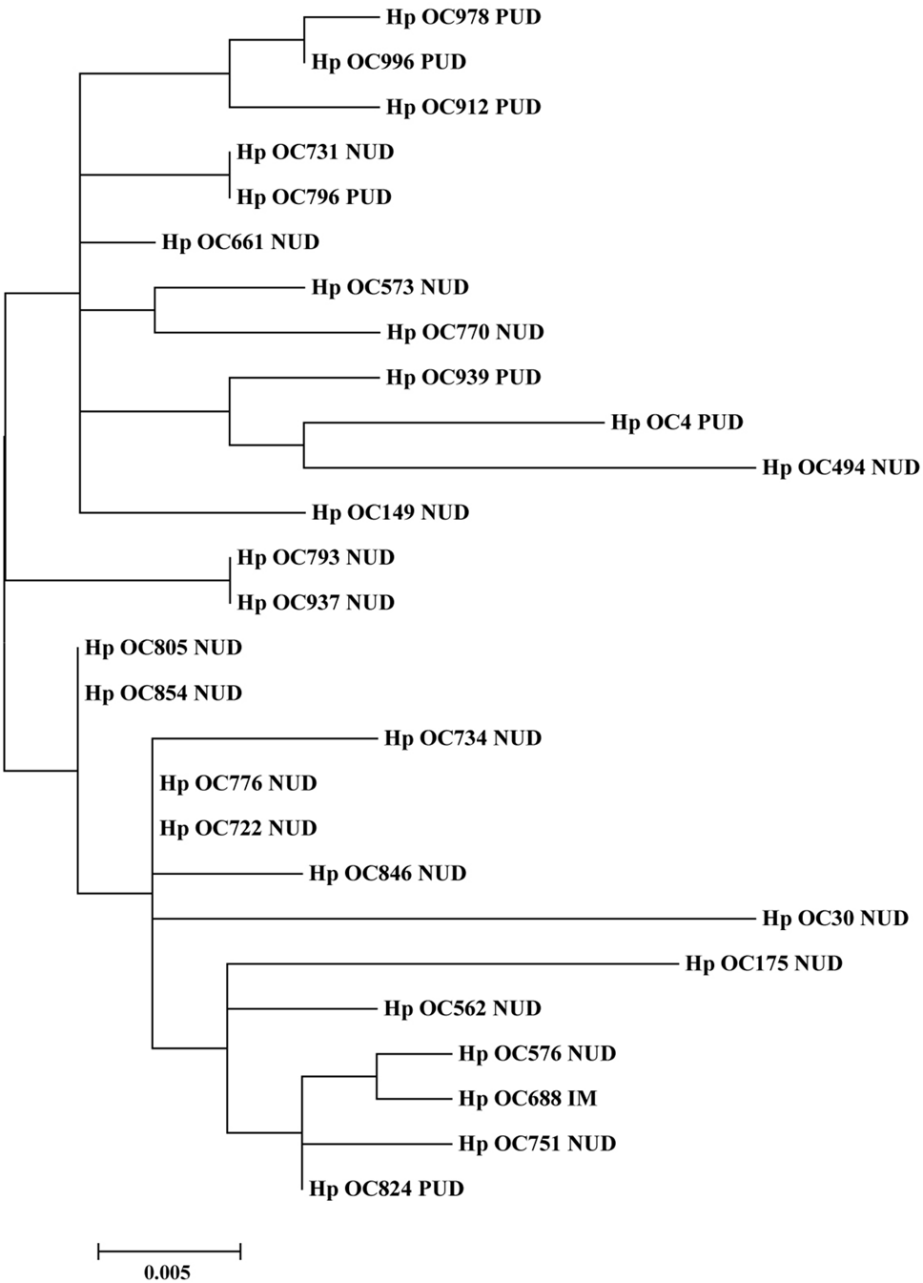
Phylogenetic tree of *H. pylori* clinical strains (*n* = 27) based on translated CagI amino acid sequences. The maximum likelihood tree of concatenated sequences was constructed using MEGA7 software with the bootstrap method at 1000 replications. The evolutionary distances were computed using the Poisson correction method.

**Figure 3:**
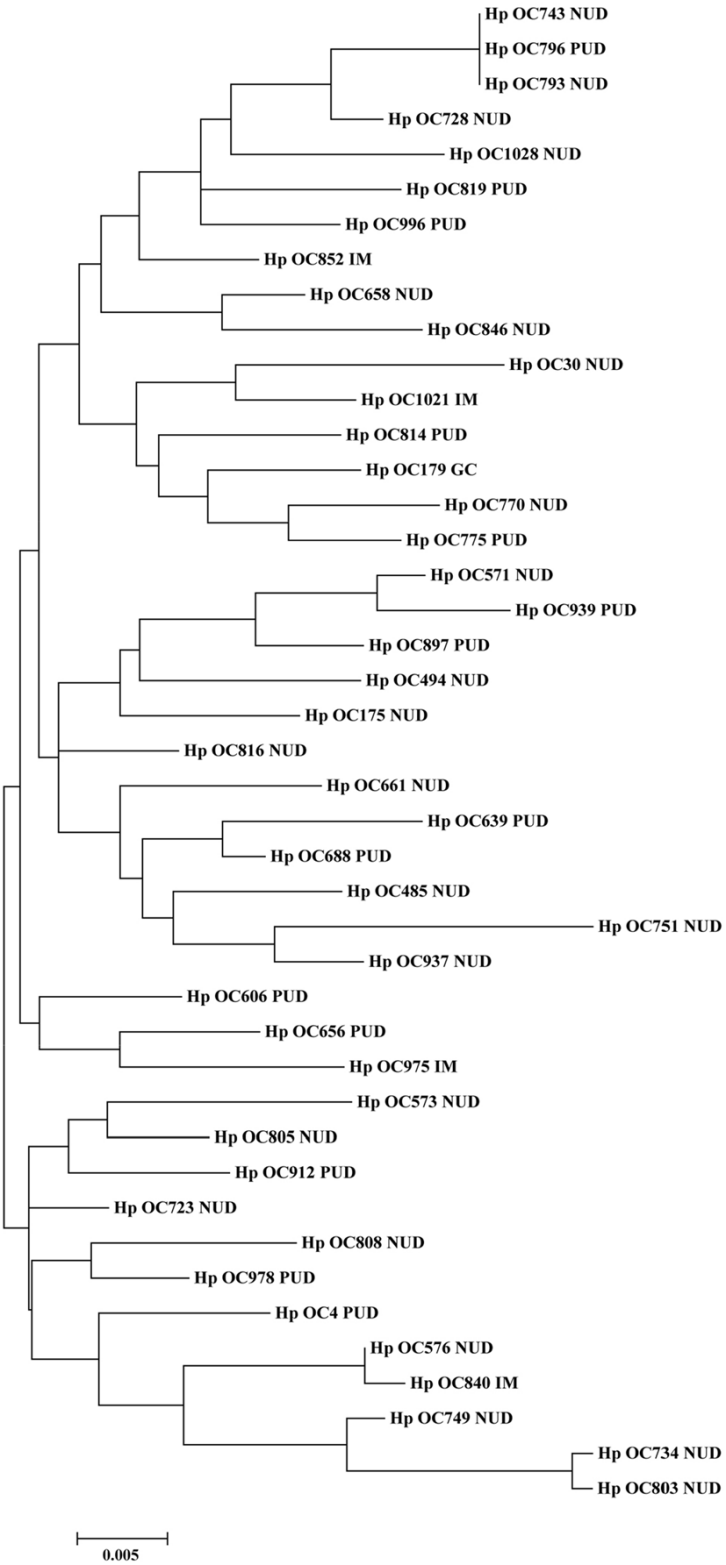
Phylogenetic tree of *H. pylori* clinical strains (*n* = 43) based on *cagN* nucleotide sequences. The maximum likelihood tree of concatenated sequences was constructed using MEGA7 software with the bootstrap method at 1000 replications. The evolutionary distances were computed using the Tamura 3‐parameter model.

**Figure 4:**
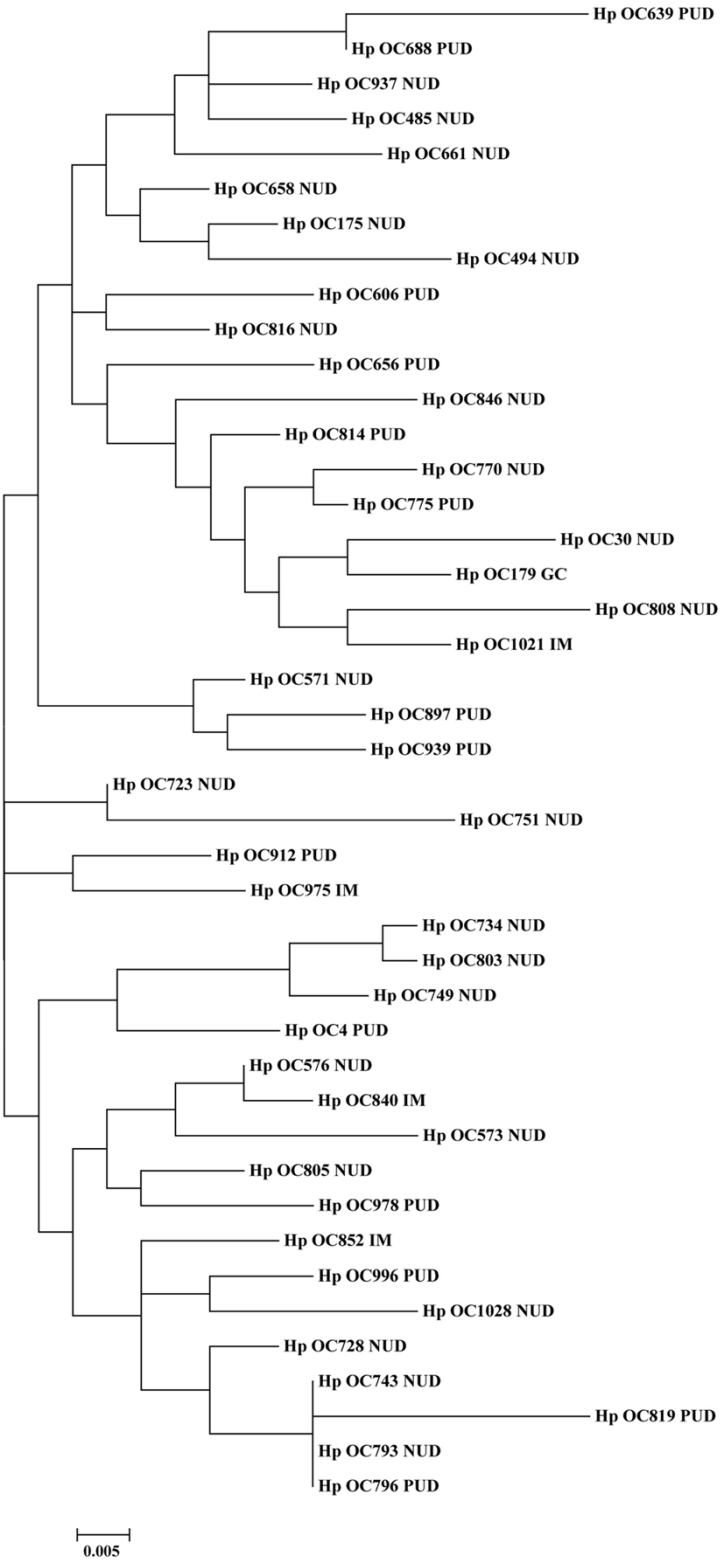
Phylogenetic tree of *H. pylori* clinical strains (*n* = 43) based on translated CagN amino acid sequences. The maximum likelihood tree of concatenated sequences was constructed using MEGA7 software with the bootstrap method at 1000 replications. The evolutionary distances were computed using the Poisson correction method.

The phylogenetic trees of *cagI* nucleotide and amino acid sequences from *H. pylori* isolates are illustrated in Figs. 1 and 2, respectively. Generally, no characteristic clusters were observed between DNA and amino acid sequences of CagI and different clinical statuses. Furthermore, based on the CagN nucleotide and amino acid sequences, phylogenetic trees were reconstructed using the Maximum Likelihood method, which are illustrated in Figs. 3 and 4, respectively. Similar to CagI sequences, the CagN phylogenetic analysis indicated no characteristic clusters concerning the clinical status.

## Discussion

Virulent *H. pylori* strains harbor the *cag*PAI (*cag*^+^) encoding a type IV secretion apparatus, which has been shown to inject CagA and possibly other virulence effectors into infected gastric epithelial cells^31^. It has been well documented that *cag*^+^ *H. pylori* strains augment the risk for severe gastritis, peptic ulceration, atrophic gastritis, dysplasia, and gastric adenocarcinoma compared to strains that lack the *cag*PAI (*cag*^−^)^32–34^. Previously, it has been described that CagI forms a functional protein complex at the bacterial cell surface by interacting with CagL, which is another important Cag secretion apparatus component. Accordingly, solid evidence suggested that CagI can interact with CagL protein and allow it to bind to integrin receptors on the target cell surface^8,17^. CagI and CagL proteins contain N-terminal signal peptides; therefore, they might be transported to the periplasm. However, these two proteins are disproportionately distributed on the bacterial cell surface^35^. Considering different views on CagI, Kumar et al.^36^ found that CagI does not participate in CagA translocation from cytoplasm to bacterial cell surface. Additionally, it has been discovered that mutation in *cagN* did not interrupt CagA delivery or IL-8 secretion and the CagN-deficient *H. pylori* strains could cause an infection similar to wild-type *H. pylori* strains. Some experiments have also indicated that CagN is not conclusively required for *H. pylori* T4SS function^16^. In another study conducted by Kutter et al. CagN was established to interact with two other *cag*PAI proteins, including CagV and CagY^35^. Thus, the biological function of CagN requires further in-depth investigation. In the current study, attempts were made to detect possible variants of CagI and CagN, as uncharacterized *cag*PAI-encoded factors, on both nucleotide and amino acid sequence levels among *H. pylori* isolates in Iran. We also investigated the distribution and variations in *H. pylori* virulence factors. Our findings revealed that 80% of *H. pylori* isolates harbored the *cagI* gene, whilst 91.4% of strains had the *cagN* gene. To the best of our knowledge, the *cagI* and *cagN* variants in *H. pylori* isolates in the subset of patients with different gastroduodenal diseases are not available in the literature. Based on our molecular findings, CagI E221 (21.0% vs. 0.0%), and V268 (26.3% vs. 14.2%) amino acid polymorphisms occurred at a higher rate in *H. pylori* isolates from NUD individuals, compared to those isolated from PUD patients. On the contrary, CagI amino acid changes G22 (42.8% vs. 5.2%), A23 (42.8% vs. 5.2%), S57 (71.4% vs. 47.3%), and S94 (85.7% vs. 52.6%) were detected at higher rates in *H. pylori* isolates from PUD patients, compared to NUD subjects.

Despite *cagN* and *cagM* being demonstrated to be conserved in the *cag*PAI throughout all *cag*^+^ *H. pylori* strains that have been sequenced so far^11^, a high level of variability in CagN nucleotide and protein sequences was observed in the present study. Furthermore, the most variable region in CagN amino acid sequence, so-called here CagNHM, was found at residues 53 to 63 and contained many missense mutations. This region is postulated to contain the GDEEITEEEKK sequence in the P12 reference strain but varied among the sequenced strains in the current study.

Our findings elucidated that there was no significant correlation between clinical diseases and *cagI* and *cagN* variants at both nucleotide and amino acid levels (*P* > 0.05), which is in accordance with a previously conducted study^25^. Pham et al. stated that the C-terminal motif (SKVIVK) in CagI is essential for T4SS function, and thus is completely conserved among *H. pylori* strains. Remarkably, the C-terminal motif of CagI is reported to be similar to the C-terminal motifs of CagL SK(I/V)IVK and CagH TKIIVK, representing the possibility that the amino acid sequences essentially act as binding motifs for a common interaction partner of all three proteins^17^. In agreement with the aforementioned study, our findings also confirmed that the CagI C-terminal motif was completely conserved among all *H. pylori* isolates^25^. Sequencing analysis of the present study also showed that a hypothetical hexapeptide motif (EAKDEN/K) was detected in residues 278–283 in CagN among 13.9% of *H. pylori* isolates. Although Bats et al. implied that the mutations and truncations in the CagN sequence were irrelevant to folding properties or the overall shape of CagN^37^, further studies are required to assess the impact of this hexapeptide motif on CagN protein structure and its role in *H. pylori* T4SS activity. Despite the alterations in various *cag* sequences, it is noticeable that all patients who had a high prevalence of *cag*PAI genes including *cagI*, *cagN*, *cagA*, and *cagL* that indicates more potential role of these genes in disease outcome.

In the present study, we further investigated the presence of various *H. pylori* virulence genotypes. In accordance with our previous studies among Iranian populations^38,39^, we detected a high prevalence of *vacA* s1 (77.1%) and *vacA* m2 (65.7%) allelic genotypes. The *vacA* s1 allele has been reported to be associated with more severe atrophic gastritis in peptic ulcer patients^40,41^. In our study, the *vacA* s1 genotype was found to be more prevalent among PUD patients; however, there was no significant association between the presence of other virulence genes and clinical disease outcomes. The mosaic combination of s- and m-region allelic genotypes also has been established to be associated with the pathogenicity of *H. pylori*^42,43^. Accordingly, type s1m1 *H. pylori* strains express large amounts of VacA toxin and are strongly associated with a higher level of inflammation and mucosal ulceration, while *vacA* s1m2-harboring strains produce a moderate amount of toxin and *vacA* s2m2 strains are virtually non-toxic and rarely associated with clinical disease^44^. A majority of *H. pylori* strains in the current study contained the *vacA* s1m2 genotype and this was mainly observed in NUD patients. On the contrary, allelic combination s1m1 or s2m2 genotypes were detected among the majority of clinical isolates of *H. pylori* in other parts of the world, and the hypervirulent *vacA* s1m1 genotype was commonly associated with PUD patients^45^. Hence, it can be inferred that the correlation between *H. pylori* genotyping and clinical outcomes of the patients varies in different geographical regions.

## Conclusion

This study investigated the diversity of *cagI* and *cagN* sequences in clinical *H. pylori* isolates from Iranian patients with different clinical diseases. We detected several putative variants of *cagI* and *cagN* sequences in *H. pylori* isolates; however, there was no significant relevance between these variants and clinical phenotypes. Our findings also demonstrated that the C-terminal SKVIVK motif within the CagI protein is conserved among all tested *H. pylori* strains. Meanwhile, the motif EAKDEN was a typical attribute identified in the C-terminal sequence of CagN protein among some of the *H. pylori* strains, which its potential impact on T4SS activity and translocation of effectors requires further in-depth investigations. Although the present study has successfully elaborated the genetic diversity of *cagI* and *cagN* genes, it has certain limitations in terms of insufficient sample size. Accordingly, exploring the possible effects of CagI and CagN variants on the T4SS activity as well as their potential interactions with other *cag*PAI components in a large number of *H. pylori* isolates appears mandatory.

## Supplementary Information

Supplementary Information.

## Data Availability

All data generated or analyzed during this study are included in this published article (and its Supplementary Information files).
