# Supplementary material for: Genetic diversity of Helicobacter pylori type IV secretion system cagI and cagN genes and their association with clinical diseases
Source: Sci Rep. 2023 Jun 24;13:10264. doi: 10.1038/s41598-023-37392-7 (PMC10290643; doi:10.1038/s41598-023-37392-7)
Supplement: Supplementary file 1 — Supplementary Information. [file 41598_2023_37392_MOESM1_ESM.pdf]

Table S1. Oligonucleotide sequences used for amplification of the *H. pylori* virulence genes of interest.

Target gene	Primer designation	Oligonucleotide sequence (5´-3´)	Annealing temperature (°C)	PCR product (bp)	Reference
16S rRNA	C97-20	GGCTATGACGGGTATCCGGC	58	764	[38]
	H3A-20	GCCGTGCAGCACCTGTTTTC			
glmM	GlmM2-F	GGATAAGCTTTTAGGGGTGTTAGGGG	56	296	[38]
	GlmM1-R	GCTTACTTTCTAACACTAACGCGC			
cagL	cagL-B4	GCAGAATTCATAACAAGCGGCTTAAAG	60	695	[38]
	cagL-B5	ATTAGAATTCATAGCCTATCGTCTCAG			
cagA	93089	AATACACCAACGCCTCCAAG	57	400	[38]
	93261	TTGTTGCCGCTTTTGCTCTC			
vacA s1/s2	VA1-F	ATGGAAATACAACAAACACAC	57	259/286	[38]
	VA1-R	CTGCTTGAATGCGCCAAAC			
vacA m1/m2	VAG-F	CAATCTGTCCAATCAAGCGAG	57	570/645	[38]
	VAG-R	GCGTCAAAATAATTCCAAGG			
babA2	bab7-F	CCAAACGAAACAAAAAGCGT	52	271	[39]
	bab7-R	GCTTGTGTAAAAGCCGTCGT			
sabA	F1-HP726-jhp663	TTTTTGTCAGCTACGCGTTC	56	487	[39]
	R1-HP725-jhp662	ACCGAAGTGATAACGGCTTG			
dupA	DupA-F	ATTCACGCCTAAGACCTCA	55	581	[39]
	DupA-R	CTGAGAAGCCTTATTATCTTGTTGG			

Table S2. Demographic data and clinical characteristics of patients colonized with *H. pylori* strains (*n*=70) in this study.

No.	Strains	Clinical status	Gender	Age (years)	*cagI* GenBank no.[a]	*cagN* GenBank no.
1	HC3	PUD	Male	46	Negative[b]	NA
2	OC4	PUD	Female	58	MG573078	MG559675
3	OC30	NUD	Male	56	MG573079	MG559676
4	HC114	PUD	Female	49	Negative	NA
5	OC149	NUD	Female	31	MG573080	NA
6	HC168	PUD	Male	49	NA[c]	NA
7	HC175	NUD	Female	27	Negative	NA
8	OC175	NUD	Female	30	MG573081	MG559677
9	OC179	GC	Female	63	NA	MG559678
10	OC180	IM	Female	39	Negative	NA
11	OC217	NUD	Female	42	Negative	Negative
12	OC235	PUD	Female	75	NA	NA
13	OC245	IM	Male	48	NA	Negative
14	OC250	PUD	Male	57	NA	NA
15	OC485	NUD	Male	54	NA	MG559679
16	OC494	NUD	Female	42	MG573082	MG559680
17	OC505	NUD	Female	39	NA	NA
18	OC557	PUD	Female	50	Negative	Negative
19	OC562	NUD	Female	43	MG573083	NA
20	OC571	NUD	Female	49	Negative	MG559681
21	OC573	NUD	Female	36	MG573084	MG559682
22	OC576	NUD	Female	42	MG573085	MG559683
23	OC606	PUD	Male	60	NA	MG559684
24	OC639	PUD	Male	25	Negative	MG559685
25	OC656	PUD	Male	41	NA	MG559686
26	OC658	NUD	Female	33	NA	MG559687
27	OC661	NUD	Male	33	MG573086	MG559688
28	OC688	IM	Female	42	MG573087	MG559689
29	OC722	NUD	Female	43	MG573088	MG559718
30	OC723	NUD	Male	47	NA	MG559690
31	OC728	NUD	Female	23	NA	MG559691
32	OC731	NUD	Female	24	MG573089	NA
33	OC734	NUD	Male	50	MG573090	MG559692
34	OC743	NUD	Male	60	Negative	MG559693
35	OC749	NUD	Male	70	NA	MG559694
36	OC751	NUD	Female	44	MG573091	MG559695
37	OC770	NUD	Female	73	MG573092	MG559696
38	OC775	PUD	Female	39	NA	MG559697
39	OC776	NUD	Male	34	MG573093	NA
40	OC785	NUD	Male	60	Negative	MG559719
41	OC790	NUD	Male	26	NA	MG559720
42	OC793	NUD	Female	41	MG573094	MG559698
43	OC796	PUD	Female	51	MG573095	MG559699
44	OC797	IM	Female	28	NA	NA

45	OC803	NUD	Male	52	NA	MG559700
46	OC805	NUD	Female	48	MG573096	MG559701
47	OC808	NUD	Female	65	NA	MG559702
48	OC810	NUD	Female	53	NA	NA
49	OC814	PUD	Female	25	NA	MG559703
50	OC815	NUD	Female	34	MG573105	Negative
51	OC816	NUD	Male	14	NA	MG559704
52	OC819	PUD	Female	32	NA	MG559705
53	OC824	PUD	Female	43	MG573097	NA
54	OC840	IM	Male	54	NA	MG559706
55	OC846	NUD	Female	52	MG573098	MG559707
56	OC852	IM	Male	45	NA	MG559708
57	OC854	NUD	Female	71	MG573099	Negative
58	OC884	NUD	Female	60	Negative	Negative
59	OC897	PUD	Female	60	Negative	MG559709
60	OC912	PUD	Female	64	MG573100	MG559710
61	OC913	PUD	Male	42	MG573106	NA
62	OC937	NUD	Female	48	MG573101	MG559711
63	OC939	PUD	Male	54	MG573102	MG559712
64	OC975	IM	Female	31	MG573107	MG559713
65	OC978	PUD	Female	45	MG573103	MG559714
66	OC983	PUD	Female	55	Negative	NA
67	OC996	PUD	Female	52	MG573104	MG559715
68	OC1021	PUD	Female	50	NA	MG559716
69	OC1028	NUD	Female	27	Negative	MG559717
70	OC1031	NUD	Female	52	NA	NA

GC, gastric cancer; IM, intestinal metaplasia; NUD, nonulcer dyspepsia; PUD, peptic ulcer disease; NA, not assigned.

[a]The accession numbers are deposited in GenBank database for *cagI* and *cagN* gene sequences of the *H. pylori* strains in this study.

[b]The *cagI*-negative or *cagN*-negative *H. pylori* strains.

[c]The *cagI*-positive or *cagN*-positive *H. pylori* strains that either were not sequenced or sequenced unsuccessfully.

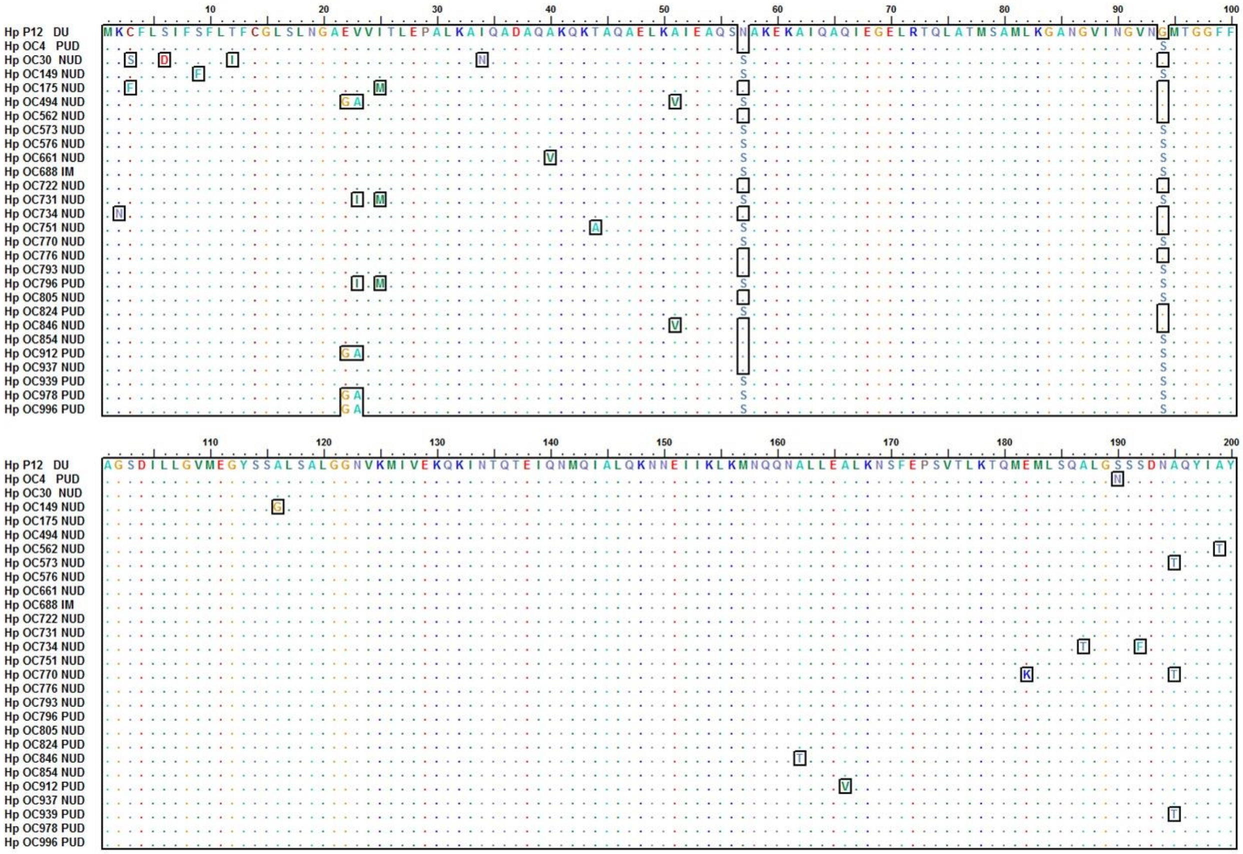
Hp P12 DU
Hp OC4 PUD
Hp OC30 NUD
Hp OC149 NUD
Hp OC175 NUD
Hp OC494 NUD
Hp OC562 NUD
Hp OC573 NUD
Hp OC576 NUD
Hp OC661 NUD
Hp OC688 IM
Hp OC722 NUD
Hp OC731 NUD
Hp OC734 NUD
Hp OC751 NUD
Hp OC770 NUD
Hp OC776 NUD
Hp OC793 NUD
Hp OC796 PUD
Hp OC805 NUD
Hp OC824 PUD
Hp OC846 NUD
Hp OC854 NUD
Hp OC912 PUD
Hp OC937 NUD
Hp OC939 PUD
Hp OC978 PUD
Hp OC996 PUD

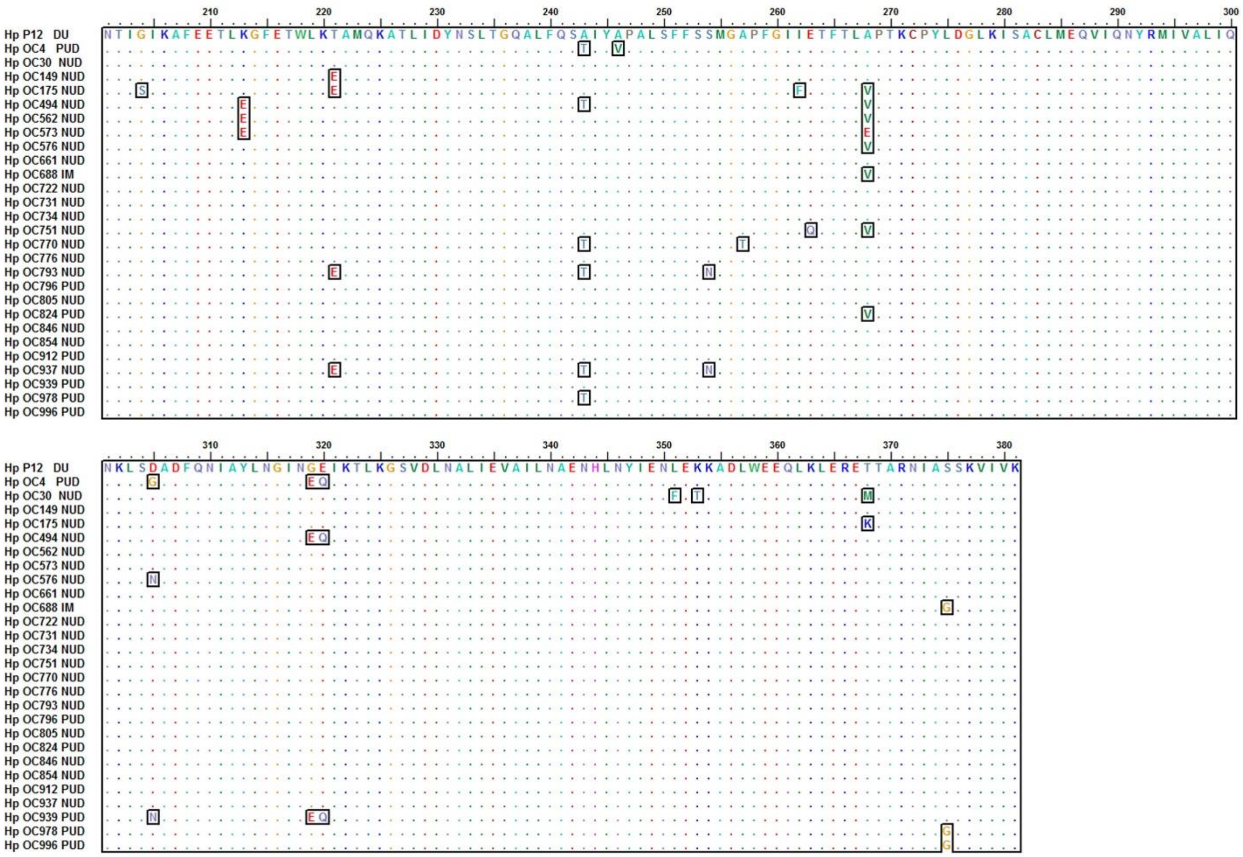

Fig. S1. Complete alignment of CagI sequences among *H. pylori* strains (*n*=27) from patients with different clinical statuses. The amino acid sequences were compared with the CagI sequences of *H. pylori* strain P12 (shown on the top line), as a reference strain. The variable and infrequent amino acid residues are surrounded by black borders. Notably, the C-terminal hexapeptide motif consisting of the SKVIVK sequence was highly conserved among the strains.

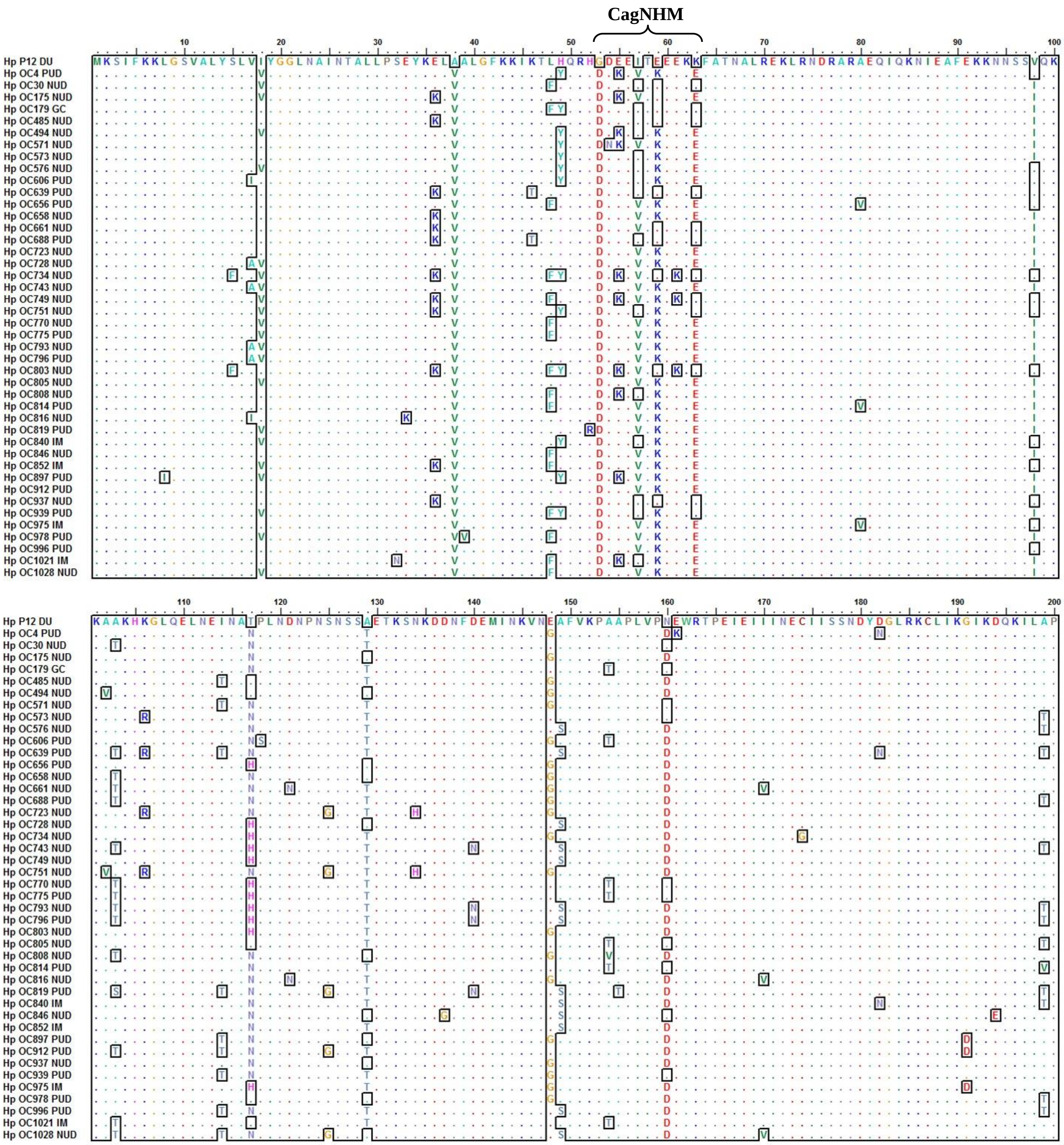
CagNHM
Hp P12 DU
Hp OC4 PUD
Hp OC30 NUD
Hp OC175 NUD
Hp OC179 GC
Hp OC485 NUD
Hp OC494 NUD
Hp OC571 NUD
Hp OC573 NUD
Hp OC576 NUD
Hp OC606 PUD
Hp OC639 PUD
Hp OC656 PUD
Hp OC658 NUD
Hp OC661 NUD
Hp OC688 PUD
Hp OC723 NUD
Hp OC728 NUD
Hp OC734 NUD
Hp OC743 NUD
Hp OC749 NUD
Hp OC751 NUD
Hp OC770 NUD
Hp OC775 PUD
Hp OC793 NUD
Hp OC796 PUD
Hp OC803 NUD
Hp OC805 NUD
Hp OC808 NUD
Hp OC814 PUD
Hp OC816 NUD
Hp OC819 PUD
Hp OC840 IM
Hp OC846 NUD
Hp OC852 IM
Hp OC897 PUD
Hp OC912 PUD
Hp OC937 NUD
Hp OC939 PUD
Hp OC975 IM
Hp OC978 PUD
Hp OC996 PUD
Hp OC1021 IM
Hp OC1028 NUD

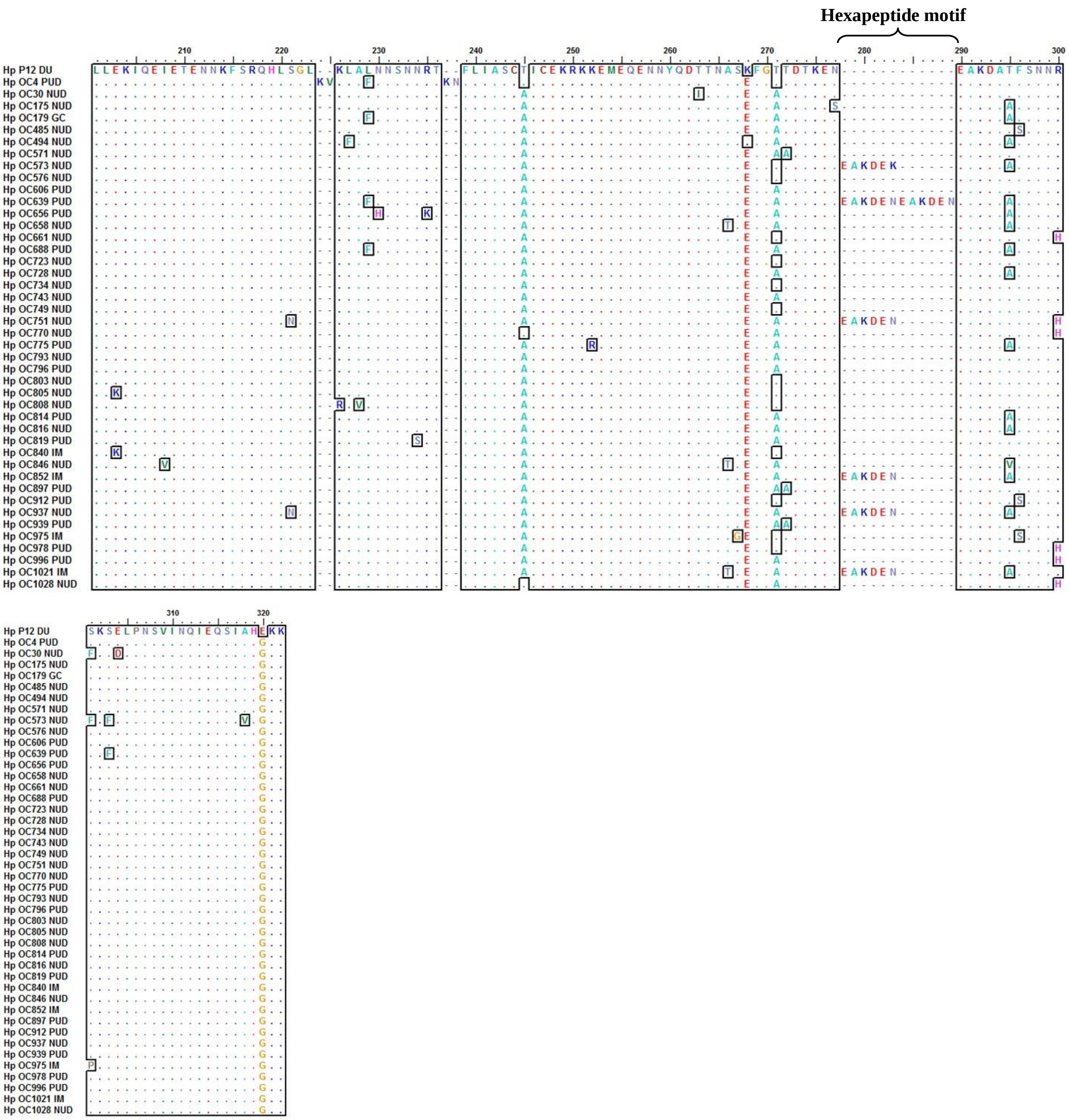

Fig. S2. Complete alignment of CagN sequences among 43 *H. pylori* strains from patients with different clinical statuess. The amino acid sequences were compared with the CagN sequences of *H. pylori* strain P12 (shown on the top line), as a reference strain. The variable and infrequent amino acid residues are surrounded by black borders. Notably, the CagN hypervariable motif (CagNHM) and the hypothetical hexapeptide motif (EAKDEN/K) are indicated at residues 53-63 and 278-283, respectively.
